# 

**Published:** 1892-11

**Authors:** 


### HALL'S JOURNAL OF HEALTH

ESTABLISHED 1854

CONTENTS.

Vol. 39. No. 11.

| | PAGE. |
|---|---|
| Fashion | 241 |
| School Cramming | 243 |
| Natural Sleep | 245 |
| A 1,000 Feet Under Ground | 247 |
| A Cool Draught of Water | 250 |
| A Human Magnet | 251 |
| Nature Makes Glass | 253 |
| Progress in Photography | 254 |
| Telescopes | 254 |
| The Insect World | 255 |
| A Universal Curse | 256 |
| Thomas Paine | 257 |
| Single Beds | 257 |
| When Forks Came In | 258 |
| The Cigarette Evil | 258 |
| Walking for Health | 259 |
| Beer in the United States | 260 |
| Miscellaneous | 260 |
| Literary | 262 |
| Whittier | 264 |

INDEX TO ADVERTISEMENTS OF HALF A PAGE AND OVER.

| | PAGE. |
|---|---|
| Dr. Jaegers | I |
| Christian Life | I |
| Magic Fuel Co. | II |
| The Rip Van Winkle Rocker | IV |
| Hall's Journal of Health | IV |
| Ayer's Cathartic Pills | V |
| Hall's Journal Premiums. | VII |
| Washington Improvement Co. | VIII |
| Herba Vita | X |
| Royal Baking Powder | XI |
| Buffalo Lithia Water | XI |

